# Activated Src requires Cadherin-11, Rac, and gp130 for Stat3 activation and survival of mouse Balb/c3T3 fibroblasts

**DOI:** 10.1038/s41417-022-00462-5

**Published:** 2022-04-11

**Authors:** Hanad Adan, Stephanie Guy, Rozanne Arulanandam, Mulu Geletu, Juliet Daniel, Leda Raptis

**Affiliations:** 1 Department of Pathology and Molecular Medicine, Queen’s University, Kingston, ON K7L 3N6 Canada; 2 Ottawa Hospital Research Institute, University of Ottawa, Ottawa, ON K1N 6N5 Canada; 3 Department of Chemistry, University of Toronto, Mississauga, ON L5L 1C6 Canada; 4 Department of Biology, McMaster University, Hamilton, ON L8S 4L8 Canada; 5 Department of Biomedical and Molecular Sciences, Queen’s University, Kingston, ON K7L 3N6 Canada; 6 Present Address: Department of Biology, McMaster University, Hamilton, ON L8S 4L8 Canada

**Keywords:** Oncogenes, Cell biology

## Abstract

We previously demonstrated that engagement of cadherins, cell to cell adhesion molecules, triggers a dramatic increase in levels and activity of the Rac/Cdc42 small GTPases, which is followed by secretion of IL6 family cytokines and activation of their common receptor, gp130, in an autocrine manner. This results in phosphorylation of the Signal Transducer and Activator of Transcription-3 (Stat3) on tyrosine-705, which then dimerizes, migrates to the nucleus, and activates transcription of genes involved in cell division and survival. In the present report we demonstrate that, in mouse Balb/c3T3 fibroblasts, mutationally activated Src^527F^ also increases Rac levels, leading to secretion of IL6 family cytokines and gp130 activation, which triggers the Stat3-ptyr705 increase. Interestingly, our results also demonstrate that cadherin-11 is required to preserve gp130 levels for IL6 family signaling. At the same time, however, activated Src^527F^ downregulates cadherin-11, in a quantitative manner. As a result, Src^527F^ expression to intermediate levels allows sufficient cadherin-11, hence gp130 levels for Stat3 activation, as expected. However, expressed to high levels, Src^527F^ eliminates cadherin-11, hence gp130 signaling, thus abolishing Stat3-ptyr705 stimulation. Taken together, these data establish for the first time a loop between Src, cadherin-11, gp130, and Stat3 activation. This fine balance between Src^527F^ and cadherin-11 levels which is required for Stat3 activation and cellular survival could have significant therapeutic implications.

## Introduction

The signal transducer and activator of transcription-3 (Stat3) is a latent cytoplasmic transcription factor. Stat3 is activated by receptor tyrosine kinases such as EGFR, cytokine receptors such as the Interleukin-6 receptor (IL6R), and non-receptor tyrosine kinases such as Src. Ligand engagement leads to phosphorylation of specific tyrosine residues on the receptors, which provide docking sites for the Src homology 2 (SH2) domain of Stat3. Thus, Stat3 is recruited to the activated receptors and, in turn, becomes phosphorylated by the receptor itself, or by associated Janus kinase (JAK) or Src family kinases [1, 2]. Following phosphorylation of the critical tyrosine-705 residue (ptyr705), Stat3 monomers associate with each other through reciprocal SH2-ptyr705 interactions to form dimers that translocate to the nucleus. There, Stat3 dimers bind DNA sequences to activate the transcription of specific genes involved in cell division and survival, such as myc, bcl-xL, mcl-1, and *survivin* while they downregulate the tumor suppressor p53 [3], thus protecting tumor cells from apoptosis [4, 5]. Hyperactivation of Stat3 is present in a large number of cancers and has been reported to be required for tumor cell growth and survival, as well as angiogenesis, metastasis, and immune evasion [6]. The fact that a constitutively active form of Stat3 (Stat3C) alone is sufficient to induce transformation of cultured fibroblasts [7] and epithelial cells [8] points to an etiological role of Stat3 in oncogenesis.

We and others previously demonstrated that engagement of cadherins, cell to cell adhesion molecules [E-, N-cadherin, or cadherin-11 (Cad11)], as occurs with a confluence of cultured cells, causes a dramatic increase in Stat3 activity in breast carcinoma lines as well as normal or transformed epithelial cells and fibroblasts [8–10]. This is triggered by a dramatic increase in protein levels and activity of the Rac1 (Rac) and Cdc42 small GTPases [11], which, in turn, causes a transcriptional upregulation of IL6 family cytokines, thus activating Stat3 through the common receptor subunit of the family, gp130, and JAK kinases [8, 12].

The Src family of non-receptor tyrosine kinases is often hyperactive in a variety of cancers [13], and activated Src is frequently associated with worse patient survival [14]. Early results demonstrated that Src activates Stat3 in cultured cells and that transcriptionally active, tyrosine-705-phosphorylated Stat3 is required for neoplastic transformation by the Src oncogene [15, 16]. Since Cad11 is also a potent activator of Stat3 [12], we revisited the question of Stat3 activation by Src, by exploring the role of Cad11 in a defined system of Balb/c3T3 cells expressing different levels of activated Src^527F^. These cells are not transformed so that, unlike tumor cells, the relationship between Src, cadherin-11, and Stat3 can be examined in the absence of confounding pathways. Our results reveal the importance of a fine balance between Src^527F^ and Cad11 levels for Stat3 activation; expressed to high levels, Src^527F^ eliminates Cad11, which, in turn, is required for gp130 signaling and Stat3 activation. Expressed to intermediate levels, on the other hand, Src^527F^ allows sufficient residual levels of Cad11 and gp130 for Stat3 activation. Taken together, these data establish a loop between Src, Cad11, gp130, and Stat3 activation, a finding which could have significant therapeutic implications.

## Materials and methods

### Cell lines and culture techniques

The Balb/c3T3 cell line (ATCC) has been described [17]. The rat-F111 line and its mT-expressing derivatives are described in [18]. All cells were grown in Petri dishes in DMEM in a 5% CO_2_ incubator. Balb/c3T3 cells and derivatives required supplementation with 10% fetal bovine serum (PAA Laboratories, cat. #A15-751), but the rat-F111 cells and derivatives required 5% calf bovine serum. It was especially important to ensure even distribution of the cells during plating, by passing them at subconfluence and pipetting vigorously with a 9” Pasteur pipette. To reduce the variability that might be caused by nutrient depletion in post-confluent cultures, the medium was changed every 24 h. Cell confluence was estimated visually and quantitated by imaging analysis of live cells under phase contrast using a Leitz Diaplan microscope and the MCID-elite software (Imaging Research, St. Catharine’s, ON).

All lines were treated with plasmocin (Invivogen #ant-mpt-1, 10 μg/ml for 2 weeks) periodically and tested for mycoplasma contamination by DAPI staining or by PCR, as described in [19].

### Gene transduction

The pWZL-Src^527F^-hygro plasmid which encodes the constitutively active chicken Src mutant and a hygromycin resistance gene was a gift from Dr. Andrew Craig, Queen’s University and propagated by transfection in Phoenix ecotropic retroviral packaging cells using the PolyJet in vitro DNA transfection reagent (SignaGen, Frederick, MD, cat. #SL100688). Following selection, clones picked were further subcloned at least twice more to eliminate revertants and propagated at subconfluence. A number of vials of each were frozen and used for a maximum of two weeks after thawing. This was especially important for high-Src^527F^-expressing cells.

Balb-shCad11 and Balb-shRac cells have been described [12, 20]. Cad11 shRNA-encoding retroviral constructs were purchased from Open Biosystems (Huntsville, AL, cat. #RMM4530-NM_009866). For shCad11 we used 4 different sequences (Table S1), with essentially identical results. Rac shRNA was purchased from Open Biosystems (cat. #RMN 1766-97047533). Following infection, cells were selected with 0.5–1 µg/ml puromycin, and individual clones expanded into lines for further experimentation.

### Western blotting

Following electrophoresis and transfer, membranes were cut into strips based on the positions of pre-stained markers and probed for Src^pY416^ (Cell Signaling, Danvers, MA, cat. #2101), pan-Src (Cell Signaling, cat. #2109), Cadherin-11 (Cell Signaling, cat. #4442), Stat3-ptyr705 (Cell Signaling, cat. #9145, Rabbit) cleaved PARP (Cell Signaling, cat. #9544), Rac (Millipore #05-389), b-actin (Cell Signaling #3700) and secondary antibodies according to the manufacturer’s protocols. Bands were visualized using the Clarity Western ECL substrate (Bio-Rad, Hercules, CA, cat. #170-5060). Images were developed by exposing the membranes to film or by using the Azure 300 Digital imager (Azure Biosystems, Dublin, CA). Photo-shop or Corel Draw software was used for the organization of non-adjusted, original images. Quantitation was achieved by image analysis using ImageJ (U.S. NIH). In all cases, band intensities were normalized to β-actin or α-tubulin levels of the same samples. Rac activation assays were performed using GST-PAK pull-down assays with the Rac activation kit from Cytoskeleton (Denver, CO, cat. #BK035), using beads coated with glutathione-S-transferase (GST) fused to the binding domain of p21-activated kinase (PAK), followed by probing for Rac by Western blotting, as previously described [8].

Experiments were repeated at least twice and with a number of lines expressing different Src levels. Stat3-ptyr705 levels were examined at different densities spanning 100% confluence, as described in Results.

### TUNEL staining

Cells were fixed with 4% paraformaldehyde and assayed for apoptosis by terminal deoxynucleotidyl transferase dUTP nick end labeling (TUNEL) using fluorescein-coupled nucleotides, according to the manufacturer’s instructions (Roche, cat. #11684795910). Cells with apoptotic nuclei were visualized and photographed under fluorescence and phase contrast illumination.

## Results

### Src^527F^ expression reduces Cadherin-11 protein levels in mouse Balb/c3T3 fibroblasts

**Fig. 1:**
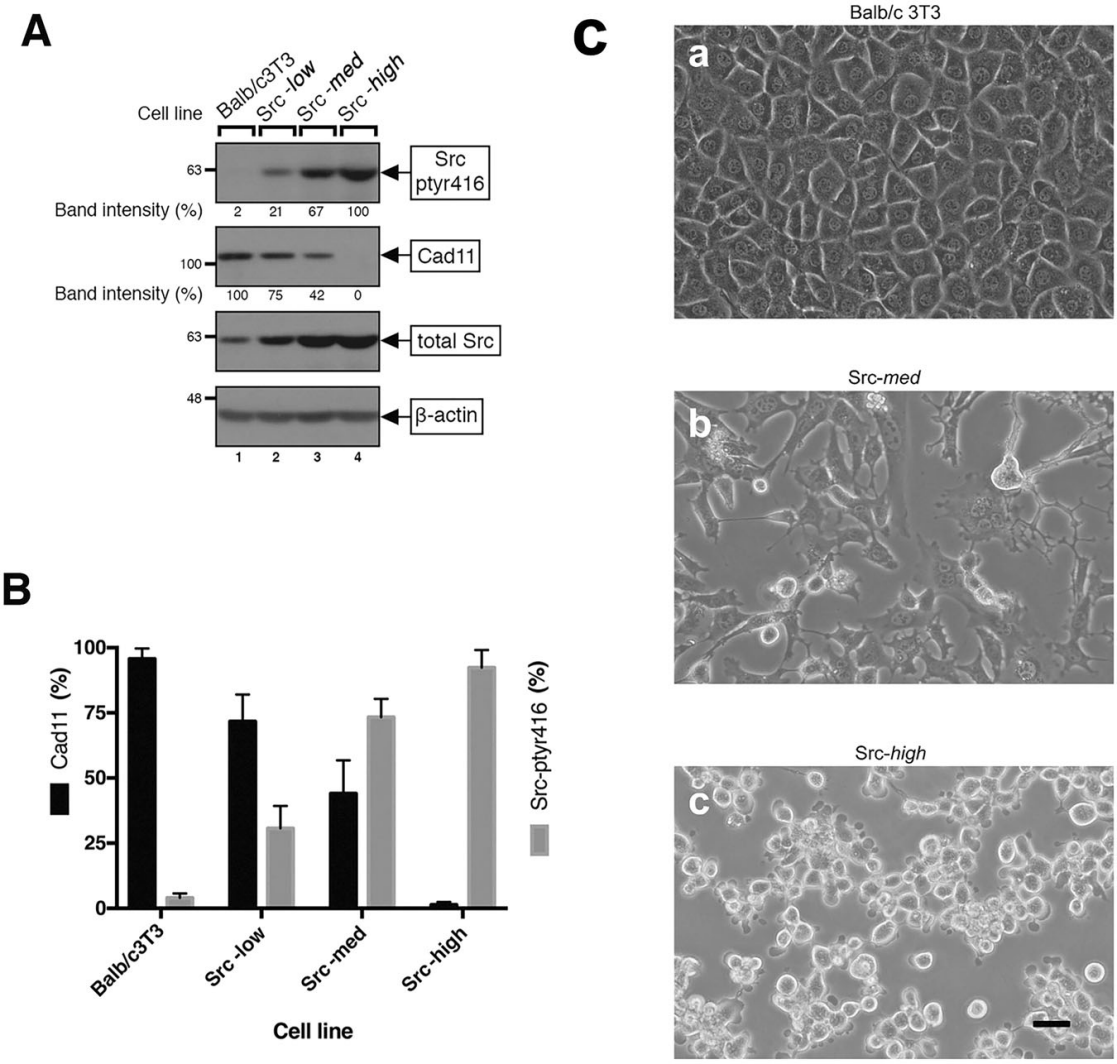
Src^527F^ expression reduces Cad11 protein levels in Balb/c3T3 cells. **A** Parental Balb/c3T3 cells (lane 1) and clones stably expressing low (Src-***low***, lane 2), medium (Src-***med***, lane 3), or high (Src-***high***, lane 4) levels of Src^527F^ were grown to 100% confluence. Detergent lysates were resolved by gel electrophoresis and probed for Src^pY416^, Cad11, pan-Src, or β-actin as a loading control, as indicated. Numbers under the lanes refer to band intensities obtained through quantitation by image analysis and normalized to β-actin levels, with the peak values of clone Src-***high*** (lane 4) for Src^pY416^, and the parental Balb/c3T3 (lane 1) for Cad11, respectively, taken as 100%. Numbers at the left refer to molecular weight markers. **B** Quantitation analysis of Cad11 and Src^pY416^ protein levels in clones in **A**. Values are averages of three independent experiments, ±SEM. **C** Morphology of Balb/c3T3 cells and clones expressing ***medium***, or ***high*** Src^527F^ levels. Cells were photographed under phase contrast illumination. Bar: 100 µm. Note the round shape of Src-*high* cells (**c**), which lack Cad11.

Src is known to negatively regulate E-cadherin expression and function [reviewed in ref. [21]]. To examine the effect of Src upon Cad11, a type II classical cadherin, constitutively active Src^527F^ was stably expressed in mouse Balb/c3T3 fibroblasts which possess high Cad11 levels [17], using a pBabeHygro-based retroviral vector and Hygromycin resistance selection (see Materials and Methods). Detergent lysates of resistant clones, together with the parental Balb/c3T3, were probed for Src^pY416^ (which correlates with Src activity) by Western blotting (supplementary Fig. S1, A, top panel). Bands were quantitated by image analysis using the value of the highest Src^Y416^- expressing clone obtained as 100%. A number of individual clones with increasing amounts of Src^527F^ were chosen for further experimentation. Results from representative clones expressing low, medium, or high Src^527F^ levels, named Src-***low***, Src-***med***, or Src-***high***, respectively, are presented in Fig. 1.

Examination of Cad11 levels in detergent cell extracts by Western blotting using the parental Balb/c3T3 as a positive control, revealed a quantitative, inverse relationship between Src^pY416^ and Cad11 levels (Fig. 1A): Src-***high*** cells had no detectable Cad11 (lane 4), Src-***med*** cells had approximately 42% the Cad11 protein levels of the parental Balb/c3T3 cells (lane 3 vs 1), while Src-***low*** cells had approximately 75% the Cad11 levels of the Balb/c3T3 (lane 2 vs 1). A number of additional clones expressing different Src^527F^ levels gave similar results (supplementary Fig. S1 B, top panel). Collectively, these findings indicate that although Src^527F^ is, in fact, able to dramatically reduce Cad11, the effect is dependent upon the levels of Src expression; elimination of detectable Cad11 required ~90% of the highest Src^527F^ levels obtained in this system.

### High Src^527F^ expression *reduces* Stat3-ptyr705 levels

**Fig. 2:**
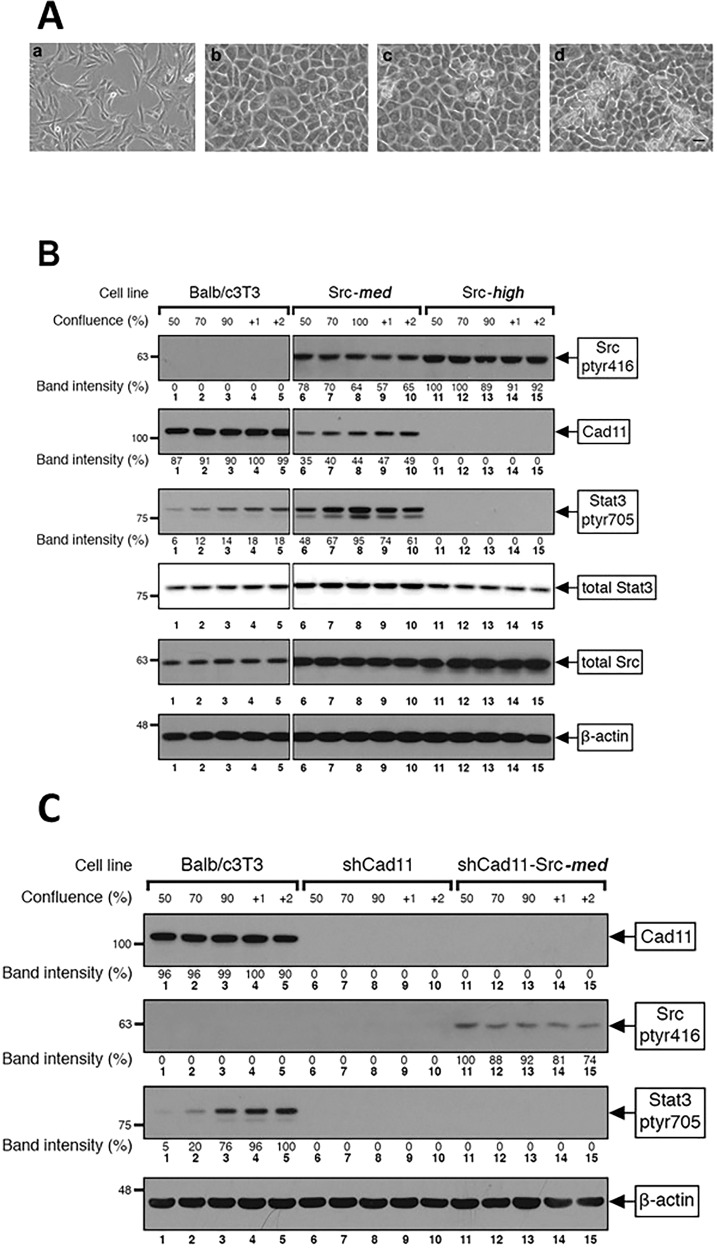
Expression of *high* Src^527F^ levels eliminates Cad11 and Stat3-ptyr705. **A** Balb/c3T3 cells grown to densities of **a** 75%, **b** 100%, **c** 100%+1 day, and **d** 100%+2 days, respectively, were photographed under phase contrast illumination. Bar: 100 µm. **B** Parental Balb/c3T3 (lanes 1–5), Src-***med*** (lanes 6–10), and Src-***high*** (lanes 11–15) cells were grown to densities of 50% to 2 days post-confluence. Detergent cell extracts were probed for Src^pY416^, Cad11, Stat3-ptyr705, total Stat3, total Src, or β-actin as a loading control, as indicated. Numbers immediately under the lanes refer to band intensities of Src^pY416^, Cad11, or Stat3-ptyr705 obtained through quantitation by image analysis and normalized to β-actin levels, with the highest values of each taken as 100%. Numbers at the left refer to molecular weight markers. Note the absence of Stat3-ptyr705 in Src-***high*** cells (lanes 11–15), despite expression of the highest Src^527F^ levels. **C** Cad11 is required for Stat3, ptyr705 phosphorylation by Src^527F^. Parental Balb/c3T3 (lanes 1–5), shCad11 (lanes 6–10), and shCad11-Src-***med*** (lanes 11–15) cells were grown to the indicated densities. Detergent cell extracts were probed for Cad11 (**A**), Src^pY416^ (**B**), Stat3-ptyr705, (**C**) or β-actin as a loading control. Numbers immediately under the lanes refer to band intensities obtained through quantitation by image analysis and normalized to β-actin levels. Numbers at the left refer to molecular weight markers.

It has been extensively documented that Src expression increases Stat3-ptyr705 phosphorylation, DNA binding, and transcriptional activity [15, 16]. As shown above, however, Src^527F^, in a quantitative manner, also downregulates Cad11, which (upon engagement) is a potent Stat3 activator. To examine this apparent paradox, we assessed the effect of different levels of Src^527F^ upon the Cad11/Stat3 axis. Since cadherin engagement, as occurs with a density of cultured cells, causes a dramatic increase in Stat3 activity [[8, 10], reviewed in ref. [22]], experiments were performed at different cell densities. Balb/c3T3, Src-***med****,* and Src-***high*** cells were plated in plastic culture dishes and, when 50% confluent and over several days thereafter (Fig. 2A), detergent cell extracts were probed for the tyr705 phosphorylated, i.e., the activated form of Stat3 by western blotting. As shown in Fig. 2B, the levels of Stat3-ptyr705 increased with density in the parental Balb/c3T3 (lanes 1–5), as previously documented [12]. Levels of Stat3-ptyr705 also increased with activated Src^527F^ expression, from the parental Balb/c3T3 (lanes 1–5) to Src-***med*** cells (Src^527F^ 67% of the highest, lanes 6–10), in agreement with previous findings [15, 16]. Interestingly, however, expression of high Src^527F^ expression (line Src-***high***) rather than increasing, dramatically decreased Stat3-ptyr705 to undetectable levels (lanes 11–15). A number of additional clones expressing graded Src^527F^ levels gave similar results (Fig. S1, B, bottom panel). Levels of the prominent Stat3 target, survivin, mirrored the levels of Stat3-ptyr705 (Fig. S1, C).

Total Stat3 was slightly reduced with a reduction in Stat3-ptyr705, as expected from the fact that Stat3 normally activates its own promotor [23], while levels of total Cad11 or Src remained essentially unaffected by cell density. These results demonstrate that, although Src^527F^, expressed to levels of up to ~60% of the maximum, does increase Stat3-ptyr705, as previously established [15, 16], expressed to high levels Src^527F^ eliminates Stat3-ptyr705. In fact, Stat3-ptyr705 was undetectable in Src-***high*** cells, i.e., dramatically lower than in the parental Balb/c3T3.

### Cadherin-11 is required for Stat3 activation by Src^527F^

The fact that the potent Stat3 activator Cad11 was reduced to undetectable levels in Src***-high*** cells along with Stat3-ptyr705, raises the question of whether Cad11 might, in fact, be needed for Stat3 activation by Src. Therefore, to explore the Cad11 requirement for the Src^527F^-mediated Stat3-tyr705 phosphorylation, we assessed the ability of Src^527F^ to activate Stat3 in Balb/c3T3 derivatives where Cad11 was knocked down through stable expression of shCad11 [12] (line shCad11). As shown in Fig. 2C, shCad11 cells expressed very low Cad11 levels (lanes 6–10). Src^527F^ was subsequently expressed in shCad11 cells to medium levels (Fig. S1 A, bottom panel, and B top panel), and Stat3-ptyr705 levels were assessed at different densities. The results revealed, as expected, a distinct increase in Stat3-ptyr705 levels with density in the parental Balb/c3T3 cells (Fig. 2C, lanes 1–5). Downregulation of Cad11 resulted in a dramatic reduction in Stat3-ptyr705 levels, at all densities examined (lanes 6–10), consistent with previous data [12]. Interestingly, however, expression of Src^527F^ in shCad11 cells did ***not*** trigger an increase in Stat3-ptyr705 levels (lanes 11–15), indicating that although Src^527F^ can activate Stat3 in the parental Balb/c3T3 when expressed to medium levels (Fig. 2B, lanes 6–10), Src^527F^ is unable to do so in the face of a Cad11 deficiency. Similarly, the converse experiment, ie expression of shCad11 in Src-***med*** cells, showed reduced Stat3-ptyr705 levels (Fig. S2). Taken together, the above findings indicate that Cad11 is required for Stat3-tyr705 phosphorylation triggered by activated Src^527F^ expression.

### Src^527F^ increases Rac protein levels and requires Rac for Stat3, ptyr705 phosphorylation

We previously demonstrated that Cadherin engagement in both epithelial cells (E-cadherin) [8] and fibroblasts (Cad11) [12] leads to a dramatic increase in Rac1 (Rac) and Cdc42 protein and activity levels, through inhibition of proteasomal degradation [24]. This triggers the transcriptional activation of the IL6 family of cytokines, hence the activation of Stat3 in an autocrine manner [22].

**Fig. 3:**
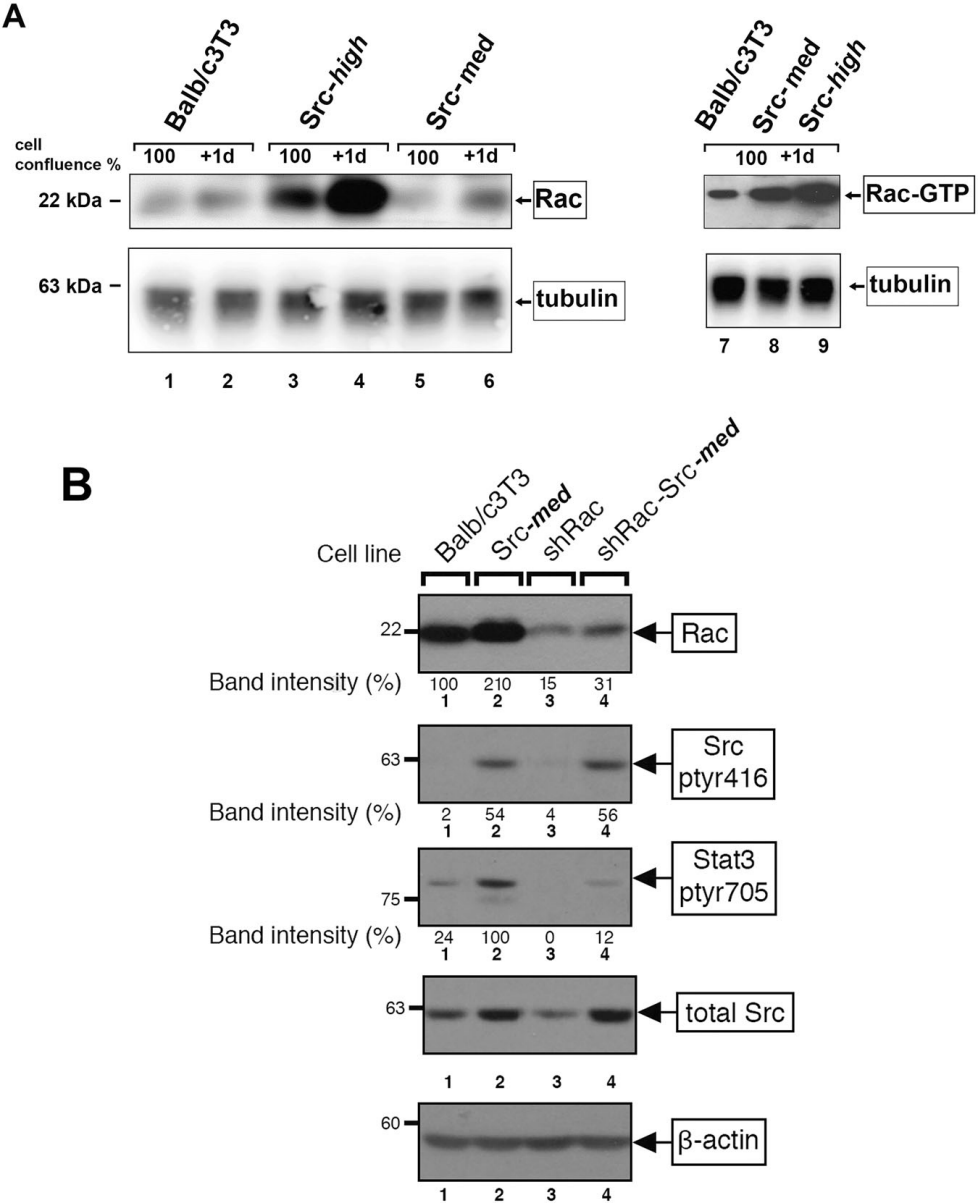
Src^527F^ increases Rac levels and activity, while Rac is required for Stat3 activation by Src^527F^. **A** Src^527F^ expression increases Rac protein levels and activity. Detergent lysates from Balb/c3T3, Src-***high****,* or Src-***med*** cells were probed for Rac protein at 100% confluence or at 100% plus one day, as indicated. Parallel lysates were subjected to Rac activity assays (Rac-GTP) as previously described [8]. **B** Rac is required for Stat3, ptyr705 phosphorylation by Src^527F^. Parental Balb/c3T3 (lane 1), Src-***med*** (lane 2), shRac (lane 3), and shRac-Src-***med*** (lane 4) cells were grown to 2 days post confluence. Detergent cell extracts were probed for Src^pY416^, total Src, Rac, Stat3-ptyr705, or β-actin as a loading control, as indicated. Numbers under the lanes refer to band intensities obtained through quantitation by image analysis and normalization to β-actin levels. Numbers at the left refer to molecular weight markers.

Since, as shown above, Cad11 is required for Stat3 activation by Src^527F^, we explored whether Rac might actually be involved in Stat3 activation by Src^527F^ as well. At first, the effect of Src upon Rac protein levels was examined. Balb/c3T3, Src-***med***, and Src-***high*** cells were plated at densities of 100% confluence or 100% plus one day and Rac levels assessed by Western blotting. As shown in Fig. 3A, Rac protein levels increased with cell density in all three lines [22]. Interestingly, Src^527F^ expression triggered an increase in Rac protein levels, which was most prominent at high Src^527F^ levels (Src-high, lanes 3–4 vs Balb/c3T3, lanes 1–2). Activated Rac-GTP mirrored the levels of Rac protein (lanes 7–9). Taken together, these data indicate that Src^527F^ expression ***is*** able to increase Rac protein levels and activity. Moreover, this increase must be independent of any effects of Cad11, since in Src-***high*** cells Cad11 is undetectable while Rac is the highest.

We next examined the Rac requirement for Stat3 activation by Src^527F^. Rac was downregulated through the expression of shRac with a retroviral vector (see Materials and Methods). As shown in Fig. 3B, shRac expression reduced Rac levels to 15% compared to the parental Balb/c3T3 (lane 3 vs 1). This Rac reduction resulted in a dramatic reduction in Stat3-ptyr705, to undetectable levels (lane 3 vs 1), in keeping with previous reports [11]. Interestingly, Src^527F^ expression in shRac cells caused only a small (12%) increase in Stat3-ptyr705 (lane 4), compared to Src^527F^ expression in the parental Balb/c3T3 (lane 2), revealing a Rac requirement for Stat3 activation by Src^527F^. It is possible that Src may be using Cdc42 as an alternate branch to transmit the signal to gp130/Stat3, which could explain the background level of Stat3-ptyr705 (12%) in shRac-Src-***med*** cells (lane 4). Taken together, these findings indicate that Src^527F^ increases Rac levels in Balb/c3T3 fibroblasts, by a mechanism that is independent of Cad11. In addition, Rac is, in fact, required for full Stat3, ptyr705 phosphorylation following Src^527F^ expression, consistent with previous reports [25].

### Secretion of Stat3-activating cytokines by high-Src^527F^-expressing cells

**Fig. 4:**
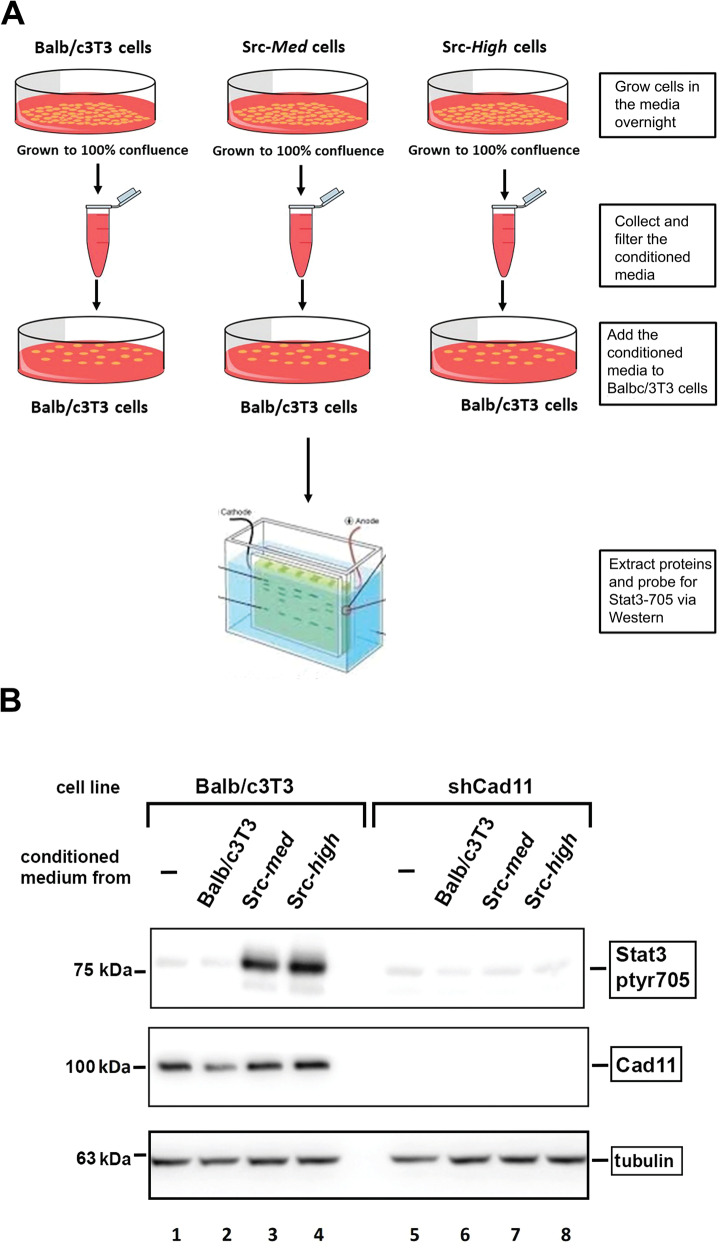
Src-*high* cells do secrete Stat3-activating cytokines. **A** Flow-sheet: medium conditioned by confluent Balb/c3T3, Src-***med*** or Src-***high*** cells (3mls serum-free DMEM medium/6 cm petri, 24 h) was collected, filtered, and added to sparsely growing, Balb/c3T3 cells for 30 min. These cells were subsequently lysed and Stat3-ptyr705 examined by western blotting. **B** Src-*high* cells do secrete Stat3-activating cytokines. Growth medium conditioned by Src-***med*** (lanes 3, 7) or Src-***high*** (lanes 4, 8) cells or the parental Balb/c3T3 (lanes 2, 6), was added to Balb/c3T3 (lanes 1–4), or shCad11 (lanes 5–8) cells growing to 50% confluence. Cells were subsequently lysed and Stat3-ptyr705 or Cad11 levels determined by Western blotting, with tubulin as a loading control. Note the dramatic increase in Stat3-ptyr705 upon treatment of Balb/c3T3 cells with medium conditioned by Src-***med*** or Src-***high*** cells (lanes 3 and 4 vs 2) and the absence of Stat3-ptyr705 increase by the same conditioned medium in shCad11 cells (lanes 7–8 vs 3, 4).

Rac was previously shown to trigger the secretion of IL6 family cytokines, through a mechanism requiring NFκB [11]. Since Rac levels are increased upon Src^527F^ expression, we assessed the secretion of cytokines by cells expressing different Src^527F^ levels. Balb/c3T3, Src-***med***, or Src-***high*** cells were grown to high densities and conditioned medium collected 24 h later. This medium was added to Balb/c3T3 cells grown to a low density (50% of confluence) and Stat3-ptyr705 levels were assessed 30 min later by Western blotting (Fig. 4A). As shown in Fig. 4B, Src-***med***-conditioned medium stimulated Stat3-ptyr705 expression in sparsely growing, Balb/c3T3 cells, substantially more than medium conditioned by the parental Balb/c3T3 (lane 3 vs 2). These results indicate that Src***-med*** cells may secrete high amounts of cytokines, which, acting in an autocrine manner, may induce the high Stat3-ptyr705 levels observed in Src-***med*** cells, as shown in Fig. 2B (lanes 6–10). Interestingly, however, medium conditioned by Src-***high*** cells also increased Stat3-ptyr705 in sparsely growing Balb/c3T3 cells, pointing to the secretion of high amounts of cytokines (lane 4), despite the fact that their own Stat3-ptyr705 levels were undetectable (Fig. 2B, lanes 11–15). Examination of IL6 levels in a conditioned medium by ELISA testing confirmed high secretion by Src-*high* cells (Fig. S4). Therefore, Src-***high*** cells, although they do secrete cytokines that are able to activate Stat3 in sparsely growing, Balb/c3T3 cells, Src-***high*** cells themselves are not able to respond to the cytokines they secrete, that is, the block to Stat3 activation by high-Src^527F^ must be at a point downstream from cytokine secretion.

To explore the possibility that the low Cad11 levels in Src-***high*** cells (Fig. 2B, lanes 11–15) may be responsible for the absence of response to the conditioned medium, the experiment was repeated by stimulating Cad11-deficient, shCad11 cells with medium conditioned by the three lines above, in a similar manner. As shown in Fig. 4B (lanes 5–8), shCad11 cells did not display any increase in Stat3-ptyr705 upon stimulation with medium conditioned by Src-***med*** or Src-***high*** cells. Taken together, the above data indicate that Src^527F^-expressing cells secrete cytokines that can activate their receptor, hence Stat3, but only under conditions where Cad11 is present.

### High Src^527F^ expression or Cadherin-11 knockdown results in gp130 and Jak-p1022/1023 downregulation

**Fig. 5:**
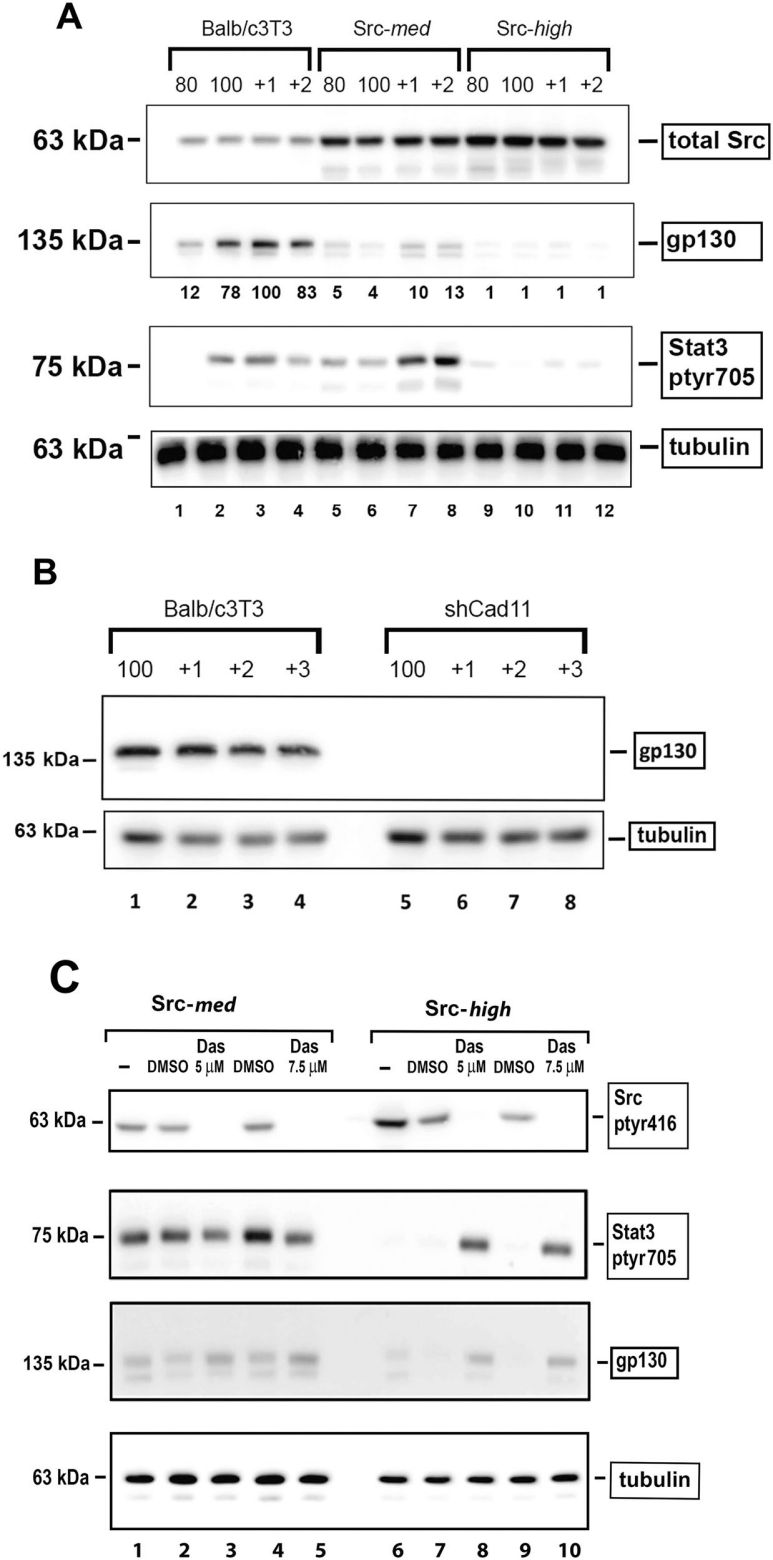
High Src^527F^ downregulates gp130 due to Cad11 downregulation, while Dasatinib restores gp130 and Stat3-ptyr705 in Src-*high* cells. **A** High Src^527F^ expression results in gp130 downregulation. Balb/c3T3 (lanes 1–4), Src-***med*** (lanes 5–8), or Src-***high*** (lanes 9–12) cells were grown to the indicated densities and detergent extracts probed for Cad11, gp130, Stat3-ptyr705, or tubulin as a loading control, as indicated. **B** Cadherin-11 knockdown results in gp130 downregulation. Balb/c3T3 (lanes 1–4) or shCad11 (lanes 5–8) cells were grown to the indicated densities and detergent extracts probed for gp130 or tubulin, as indicated. **C** Dasatinib treatment of Src-***high*** cells restores gp130 and Stat3-ptyr705 level**s** Src-***med*** (lanes 1–5) or Src-***high*** (lanes 6–10) cells grown to a density of one day after confluence were untreated (lanes 1 and 6), treated with 5 µM (lanes 3, 8) or 7.5 µM (lanes 5, 10) Dasatinib or the corresponding amounts of the DMSO carrier alone, as indicated. Detergent extracts were subsequently probed for Src-ptyr416, Stat3-ptyr705, gp130, or tubulin as a loading control, as indicated.

Cytokines of the IL6 family normally activate Stat3 following attachment to their receptor. This receptor consists of two subunits, an extracellular one which is responsible for ligand binding and is specific for each member of the family, and a common intracellular subunit, gp130. Following ligand binding, the Jak kinase is recruited to the receptor, and this is necessary for Stat3 activation [26]. Since Src-***med*** and Src-***high*** cells do secrete high amounts of IL6 family cytokines, we examined the levels of gp130 in these cells by Western blotting. As shown in Fig. 5A, expression of medium Src^527F^ levels substantially reduced gp130 protein levels (lanes 5–8 vs 1–4), while high Src expression levels eliminated gp130 altogether (lanes 9–12), concomitant with the elimination of Stat3-ptyr705. Interestingly, Stat3-ptyr705 levels were higher in Src-***med*** cells (lanes 5–8) than Balb/c3T3, despite their lower gp130 levels, presumably due to higher Rac and IL6 family cytokines secreted by Src-***med*** than the parental Balb/c3T3. However, high Src^527F^ levels that eliminated Cad11 and gp130, eliminated Stat3-ptyr705 as well (lanes 9–12). Levels of Jak1-p1022/1023 mirrored levels of gp130 and Stat3 (Fig. S3) in all three lines.

To examine whether the absence of gp130 in Src-***high*** cells could, in fact, be attributed to the absence of Cad11, we examined gp130 levels in shCad11 cells. As shown in Fig. 5B, gp130 was undetectable in shCad11 cells (lanes 5–8), pointing to the possibility that Cad11 is, in fact, required for the integrity of the gp130 receptor. Similar results were obtained with rat-F111 fibroblasts expressing the middle Tumor Antigen of polyomavirus [mT [18]], which induces transformation by binding to and activating c-Src (Fig. S7). Taken together, the above data reveal that reducing the levels of Cad11 either through the expression of high Src^527F^ levels or shRNA, results in downregulation of the gp130 receptor and this prevents Stat3 phosphorylation by Src^527F^/Jak1. It is possible that the role of Cad11 is to stabilize the gp130 receptor at the membrane, in a manner reminiscent of the E-cadherin requirement for proper activation of the gp130 receptor in mouse embryonic stem cells [27].

### Dasatinib treatment of Src-*high* cells restores gp130 and Stat3-ptyr705 levels

Despite the fact that Src has been found to be hyperactive in a number of cancers, Src inhibitors such as Dasatinib were found to be largely ineffective in clinical trials [28, 29]. It is certainly possible that other, Src-independent pathways were active in these tumors. However, a fundamental inability of Src inhibitors to reduce Stat3 activity in cells expressing high Src activity cannot be excluded. To explore the possibility that an increase (rather than decrease) in Stat3 activity upon Src inhibition might explain this apparent paradox, Src***-med*** and Src-***high*** cells were treated with the inhibitor Dasatinib or the DMSO carrier and detergent lysates probed for gp130 and Stat3-ptyr705. As shown in Fig. 5C, Dasatinib treatment of Src-*med* cells caused a slight increase in gp130 (lanes 3 and 5 vs 2 and 4) above the DMSO controls. Interestingly, however, treatment of Src-*high* cells with Dasatinib caused a prominent gp130 increase, which translated into a robust increase in Stat3-ptyr705 (lanes 8 and 10 vs 7 and 9). These results confirm that expressed to high levels, Src actually reduces gp130 and Stat3 activity. Thus, the exact level of Src expression in a given cancer could offer a potential explanation for the inconsistent results of Dasatinib or other Src inhibitors in clinical trials; treatment of high-Src cancers would actually increase Stat3 activity, leading to metastasis.

### Cadherin-11 and Rac are required for survival of Src^527F^-expressing cells

**Fig. 6:**
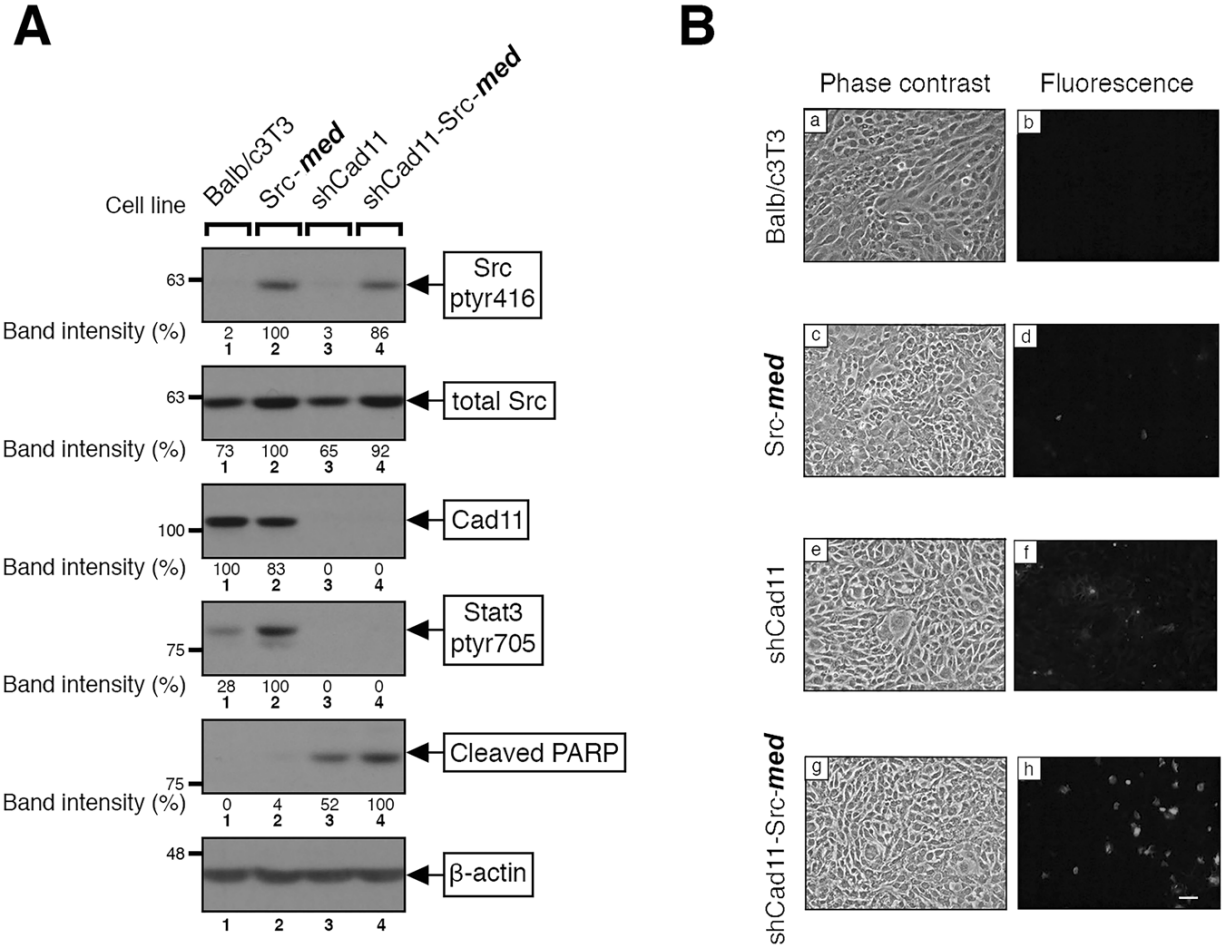
Expression of Src^527F^ in shCad11 cells promotes apoptosis. **A** Parental Balb/c3T3 (lane 1), Src-***med*** (lane 2), shCad11 (lane 3), and shCad11-Src***-med*** (lane 4) cells were grown to 1 day post-confluence. Detergent cell extracts were probed for Src^pY416^, Cad11, Stat3-ptyr705, cleaved PARP, total Src or β-actin as a loading control, as indicated. Numbers under the lanes of the upper panels refer to band intensities obtained through quantitation by image analysis and normalized to β-actin levels, with the peak values of Src-***med*** (lane 2) for Src^pY416^ and Stat3-ptyr705, Balb/c3T3 (lane 1) for Cad11, and shCad11-Src (lane 4) for cleaved PARP taken as 100%, respectively. Numbers at the left refer to molecular weight markers. **B** Balb/c3T3 (**a**, **b**), Src-***med*** (**c**, **d**), shCad11 (**e**, **f**), and shCad11-Src***-med*** (**g**, **h**) cells were grown to 1 day post confluence and apoptosis examined by TUNEL staining (see Materials and Methods). Cells were photographed under phase contrast and fluorescence illumination. Bar: 100 µm.

**Fig. 7:**
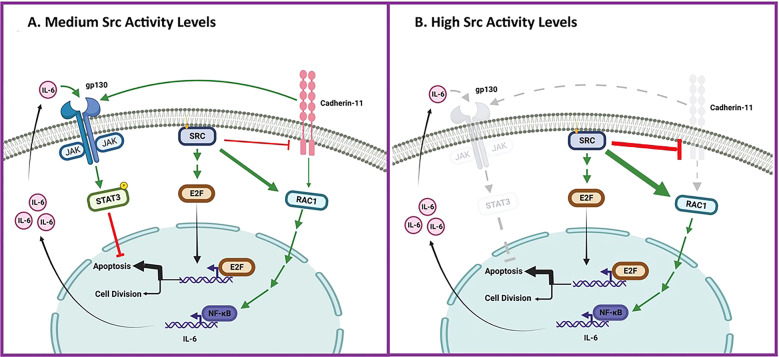
Cad11 is required for Src-mediated Stat3 activation and cellular survival. **A** Medium Src activity levels: activated Src increases Rac levels and activity in a Cad11-independent manner. This leads to transcriptional upregulation of IL6 family cytokines, followed by activation of the gp130 receptor subunit, then Jak and Stat3. Although Src at the same time downregulates Cad11, which is required for gp130 function, the residual gp130 levels are sufficient to allow Stat3, ptyr705 phosphorylation, and activation. At the same time, Src^527F^, through the Ras/Raf/Erk/Rb pathway activates the transcription factor E2F which leads to apoptosis. However, Stat3 inhibits the apoptosis triggered by high E2F activity, with cell proliferation as a result. **B** High Src activity levels: paradoxically perhaps, upon high Src^527F^ expression (or upon genetic downregulation of Cad11), Cad11 levels are reduced to non-detectable and this leads to gp130 downregulation, with a dramatic reduction in Jak and Stat3 activity, so that cells succumb to apoptosis as a result. Engagement of Cad11 also increases Rac protein levels and activity, independent of Src [8].

Previous results have shown that Stat3 induces anti-apoptotic genes such as *bcl-xL, mcl-1,* and *survivin* [4, 30, 31], it downregulates the p53 promoter [3], while it can affect the cellular metabolism through its ser727-phosphorylated form, in a way that it protects tumor cells from apoptosis further [32–34]. On the other hand, Src^527F^, besides Stat3, is known to activate the E2F transcription factor family, which actually promotes apoptosis, through the Ras/Raf/Erk pathway [35]. Therefore, the question arises as to the net effect of Src^527F^ expression upon apoptosis vs survival, which could be different depending upon Src activity levels. To answer this question, cells were grown to 1 day post-confluence and apoptosis examined by TUNEL staining and analysis of poly (ADP-ribose) polymerase (PARP) cleavage by Western blotting. As shown in Fig. 6B, Src-***med*** cells displayed a very low level of apoptosis compared to the parental Balb/c3T3 (Fig. 6B, b vs d), suggesting that the pro-apoptotic effect of E2F activation by Src^527F^ may be prevailing over the anti-apoptotic effect of Stat3 (Fig. 7).

We next examined the role of Cad11 in the survival of Src^527F^-expressing cells; as shown in Fig. 6A, shCad11 cells had a notably higher level of cleaved PARP compared to Balb/c3T3 (Fig. 6A, lanes 3 vs 1) and higher TUNEL staining (Fig. 6B, panels f vs b), indicating that knockdown of Cad11 reduces Stat3-ptyr705 levels and leads to apoptosis. Interestingly, expression of Src^527F^ in shCad11 cells triggered a further increase in cleaved PARP (Fig. 6A lane 4 vs 3) and TUNEL staining (Fig. 6B, panels h vs f). Similar results were obtained when the sequence of Src^527F^ and shCad11 expression was reversed, i.e. with Src-***med*** vs Src-***med-***shCad11 cells (Supplementary data, Fig. S5). Taken together, these results indicate that Cad11 deficiency triggers apoptosis in Src^527F^ cells, that is, the Cad11/Stat3 axis is a crucial determinant of survival of Src^527F^-transformed mouse fibroblasts. These findings may explain the inability to express high Src^527F^ levels in shCad11 cells (Fig. S1 A bottom panel) since high Src^527F^ levels would promote apoptosis in shCad11 cells, presumably due to high E2F levels combined with very low Stat3-ptyr705. As expected, Src^527F^ expression triggered apoptosis in Rac-deficient, shRac cells as well (Fig. S6). Taken together, our results point to the Cad11/Rac/Stat3 axis as a crucial signaling pathway for Src-transformed cell survival.

## Discussion

Cadherin-11 is abundant in osteoblasts, and it has been demonstrated to promote metastasis primarily to the bone, at least in breast [36] and prostate [37] cancer [38, 39]. Furthermore, high Cad11 expression is associated with invasive human breast cancer [40] and poor overall survival in gastric cancer [41]. Such tumors invariably express high levels of activated Src or other oncogenes to drive the transformed phenotype [9]. Interestingly, our results now indicate that Src expression per se leads to a dramatic reduction or elimination of Cad11, the main cadherin expressed in Balb/c3T3 fibroblasts and that ***higher*** Src levels, rather than increase as expected, actually reduce, or ***eliminate*** Stat3-ptyr705 and activity. This apparent paradox raises the question of the actual role of Cad11 in the Src-induced Stat3 activation and metastasis. Therefore, we attempted to explore the following questions:

1. How can Cad11 promote the metastasis of tumor cells, where the Src oncogene downregulates or eliminates Cad11?
2. What is the role of Cad11 in Stat3 activation by Src?
3. What is the biological role of Cad11 in the survival of Src^527F^-expressing cells?

These are critical questions regarding Cad11/Stat3 in Src Biology and neoplasia.

### Activated Src^527F^ expression downregulates Cadherin-11 in a quantitative manner

Expression of activated Src was previously shown to disrupt E-cadherin engagement and the cadherin/catenin complex in both tumor cells and transformed fibroblasts [42]. This negative regulation of E-cadherin occurs through several different mechanisms, including alterations in E-cadherin turnover and expression [reviewed in [21]]. Our results now reveal that Cad11 also is negatively regulated by Src. Furthermore, by stably expressing different levels of activated Src^527F^ in Balb/c3T3 cells, we demonstrated a quantitative negative effect of Src upon Cad11 protein levels; ***low*** Src^527F^ expression, ~21% of the highest achieved, reduced Cad11 to 75% of Balb/c3T3, ***medium*** Src expression, ~67% of the highest, reduced Cad11 to 42%, while ***high*** Src levels eliminated Cad11 entirely.

The quantitative effect of Src upon Cad11 may be a result of threshold effects of some or all of the different effectors causing Cad11 downregulation. The exact mechanism is currently under investigation. Since Balb/c3T3 cells, in addition to Cad11, also express N-cadherin [12], which also activates Stat3, the apparent absence of Stat3-ptyr705 in Src-***high*** cells may be an indication that N-cadherin as well is downregulated by Src. In any event, these findings broaden the scope of known Src effectors to include mesenchymal cadherins.

Since ***high*** Src levels were found to completely eliminate Cad11, and Cad11 is required for metastasis [37, 43, 44], it appears that actual metastatic tumors cannot possibly possess such high levels of Src activity; that is, a fine balance between Src and Cad11 levels may be required for the formation of a metastatic tumor, as observed in ***medium***-Src-expressing cells, i.e. this level of Src activity may be more relevant to cancer in vivo.

### Role of Cadherin-11 in gp130 signaling and Stat3 activation by Src^527F^

Previous reports have extensively documented that Src activates Stat3 [15, 16]. Our results now further demonstrate that progressively higher Src^527F^ expression in individual clones, up to ~60% of the highest levels achieved, results in proportionally higher Stat3-ptyr705 levels, as expected. Interestingly, however, expression of Src^527F^ levels above 90% of the highest (where Cad11 was eliminated), rather than resulting in even higher Stat3-ptyr705 levels, was found for the first time to eliminate Stat3-ptyr705 entirely. The fact that Cad11 downregulation indeed prevented Stat3, ptyr705 phosphorylation by Src^527F^ (Fig. 2C), indicates that Src^527F^ requires Cad11 to achieve Stat3-ptyr705 phosphorylation, despite the fact that Src^527F^ downregulates Cad11 at the same time. This is further reinforced by the fact that Dasatinib treatment of Src-***high*** cells, rather than reducing as expected, dramatically increased Stat3-ptyr705 levels (Fig. 5C). These results also offer an explanation for the slight increase in Stat3 activity previously observed upon Src downregulation [45].

What is the role of Cad11? It was previously reported that Src requires Rac function for Stat3 activation, while Src activates Rac through the exchange factors, Tiam and Vav2 [25]. Our results now further demonstrate that Src^527F^ (in proportion to its levels) increases Rac protein levels as well. Interestingly, we previously demonstrated that Rac, upregulated either through cadherin engagement [8, 10, 12], or mutational activation (Rac^V12^) increases transcription of IL6 family cytokines which stimulate the gp130 receptor in an autocrine manner, leading to an increase in activity of Jak and Stat3 [11]. Since the Rac increase is the highest in the Src-***high*** cells (Fig. 3A), when Cad11 is absent, the Src^527F^-stimulated, Rac increase must occur in a Cad11-independent manner. Our results further show that in the absence of Cad11, either because of shCad11 knockdown, or ***high***-Src expression, gp130 levels are undetectable. That is, Cad11 is required to maintain sufficiently high gp130 levels to allow signaling from Rac to gp130, then through Jak to Stat3. This is reminiscent of a previous report [27] indicating that E-cadherin is required for proper activation of the IL6/gp130 signaling pathway in mouse Embryonal Stem cells. Therefore, it appears that at intermediate Src^527F^ levels, the residual Cad11 levels are sufficient to maintain gp130, while the total absence of Cad11 in Src-***high*** cells results in the downregulation or degradation of gp130, hence the absence of Jak and Stat3 activation. The fact that Src cannot fully activate Stat3 in gp130 KD cells as previously demonstrated [46], further reinforces the importance of gp130. That is, a fine balance between Cad11/gp130 and Src/Rac is apparently required to induce IL6 family secretion, and preserve sufficient gp130 levels to allow signaling through the Src/Rac/IL6/gp130/Jak/Stat3 axis (Fig. 7, A vs B).

Our results are consistent with earlier findings indicating that activated Src requires Jak1 function to activate Stat3, aided by receptors such as PDGFR, acting as a scaffold [47]. Since in Src-***high*** cells Stat3-ptyr705 is undetectable, it is possible that Cad11 is required for the function of other receptors such as PDGFR as well, even when they merely have a scaffolding role in Jak/Stat3 activation. It is also possible that other cadherins, such as N-cadherin which is also present in Balb/c3T3 cells [12], can function in a manner similar to Cad11 in preserving receptor function. Experiments are underway to resolve these possibilities.

Interestingly, two other signal transducers known to be activated by IL6, Erk, and Akt are not affected by cell density [12, 22]. We recently demonstrated that at low densities, engagement of integrins with the focal adhesion kinase (FAK) activates the FAK/Src complex, and this results in activation of tyrosine-kinase receptors, Erk1/2 and Akt [48]. Interestingly, the FAK/Src complex cannot activate Stat3 in the absence of Cad11 engagement (i.e., in sparsely growing cells), possibly required to maintain gp130 function.

### Role of the Cad11/Stat3 axis in Src^527F^-induced apoptosis

Since the initial discovery that Stat3 is required for transformation by activated Src [15, 16], it has become evident that Stat3 inhibition in Src-transformed cells induces apoptosis, not simply reversion of the cell to a normal phenotype. This observation provided the first clue as to the important role of Stat3 in the survival of Src-transformed cells.

A variety of receptor or non-receptor tyrosine-kinase oncogenes, trigger the activation of the E2F family of transcription factors (the “activating” E2Fs, E2F1-3a [49]), which was shown to occur in the majority of tumors. E2F activation occurs through phosphorylation and inactivation of the Rb (retinoblastoma susceptibility) family of nuclear phosphoproteins. E2F targets include genes involved in DNA synthesis as well as growth factor and receptor genes [50]. Interestingly, at the same time, E2F activates apoptosis through both p53-dependent and independent pathways [[51, 52]; reviewed in [53, 54]]. Certainly, apoptosis is normally prevented due to the activation of survival factors such as PI3k or Stat3 by tyrosine-kinase receptors induced by E2F itself, or directly by Src or other kinases which had activated E2F in the first place, so that transformation does occur. In this schema, upon inhibition of Stat3 activity, tumor cells, having high E2F levels, may succumb to apoptosis [55]. Most importantly, inhibition of Cad11 would induce apoptosis (through Stat3 inhibition) in metastatic tumor cells specifically, since normal cells would have low E2F activity, hence would be spared (Fig. 7). In fact, our results indicated that Cad11-deficient cells succumbed to apoptosis upon Src^527F^ expression; levels of cleaved PARP and TUNEL staining were higher in shCad11-Src-***med*** cells than in Src-***med*** cells which express similar levels of Src^527F^.

## Conclusion

### Targeting Cad11 in cancer

The observation that in cultured cells, and potentially in vivo, high Src levels downregulate Cad11, which, in turn, may be required for metastasis, especially to the bone, may signify that actual metastatic tumors cannot possibly express such high Src levels as to eliminate Cad11. Apparently, a fine balance between Src and Cad11 levels is required, as to allow both transformation (through Src) and metastasis (through Cad11), for the formation of a metastatic tumor to occur. As a result, pharmacological inhibition of Src would increase Cad11/gp130/Stat3 levels in tumors with high Src activity, hence increase, rather than decrease, the metastatic ability of the tumor, leading to a deterioration in clinical outcome. The poor performance of Src inhibitors including Dasatinib in clinical trials is consistent with these findings [56–59].

Our results also stress for the first time the importance of Cad11, Rac, and IL6/gp130 signaling in Src-triggered induction of Stat3, tyr705 phosphorylation, and activation. As such, Cad11 and likely other cadherins are emerging as not only powerful Stat3 activators, but also as critical mediators of Stat3 signaling from Src and possibly other oncogenes, despite the fact that, at the same time, Src downregulates cadherins. Conversely, downregulation of Stat3-ptyr705 by caveolin-1, the main protein of caveolae, has been shown to be mediated through the downregulation of Cad11 [20]. Further investigations will clarify the role of the Cad11/Rac/gp130 axis, to unearth druggable targets. Being on the cell surface, Cad11 should be especially accessible.

## Supplementary information

Supplementary Data

## Data Availability

Unfortunately, the corresponding author recently retired from her position as professor and had to clean out and close down her laboratory. Because they did not anticipate this request they did not keep any materials. They do have the original scans of western blotting experiments performed by the first author, i.e., Figs. 3A, 4, and 5, using Azure imaging technology. Unfortunately, the rest of the western blots were done using film and by co-authors that have left the laboratory several years ago, when this was not a requirement.
