# Supplementary material for: Activated Src requires Cadherin-11, Rac, and gp130 for Stat3 activation and survival of mouse Balb/c3T3 fibroblasts
Source: Cancer Gene Ther. 2022 Apr 11;29(10):1502–13. doi: 10.1038/s41417-022-00462-5 (PMC9576600; doi:10.1038/s41417-022-00462-5)
Supplement: Supplementary file 1 — Supplementary Data [file 41417_2022_462_MOESM1_ESM.pdf]

Supplementary data

Construction of cell lines stably expressing Src^{527F}

Src was expressed in mouse Balb/c3T3 fibroblasts and their shCad11- or shRac-expressing derivatives (shCad11 or shRac, respectively), using a retroviral vector. Briefly, one 24-well plate of Balb/c3T3 and two 24-well plates of shCad11 cells (2 x 10^4 cells per well) were infected with the same culture supernatant from a Phoenix packaging line secreting an ecotropic retroviral vector coding for Src^{527F} sequences and hygromycin resistance selection (see Materials and Methods). Cells in each well were passed into 6 cm plates and selected for hygromycin resistance. A number of independent clones were picked and expanded into lines. For screening, detergent cell extracts from Balb/c3T3 and shCad11 cells, before or after Src^{527F} expression, were resolved by gel electrophoresis and probed for the active, tyrosine-416-phosphorylated form of Src (Src^{pY416}; see Materials and Methods). As a loading control, the same extracts were probed for β–actin.

Results from representative clones are shown in Fig. S1A (top panel): Most hygromycin-resistant Balb/c3T3 clones obtained expressed detectable amounts of Src^{527F}. On the other hand, out of 25, shCad11 hygromycin-resistant clones, only one displayed a significant amount of Src^{527F} (Fig. S1A (bottom panel, lane 22), even though twice as many shCad11 cells had been infected with the same stock of the Src^{527F} retroviral vector.

Clones Src-7a, Src-8a and Src-19b were the highest Src^{527F} expressors. Clone Src-7a was designated as Src-***high*** and its Src^{pY416} value was taken as 100%. Clone 16b, with 21% was designated as Src-***low*** and clone 6a with 67% was designated as intermediate (Src-***med***), respectively.

A

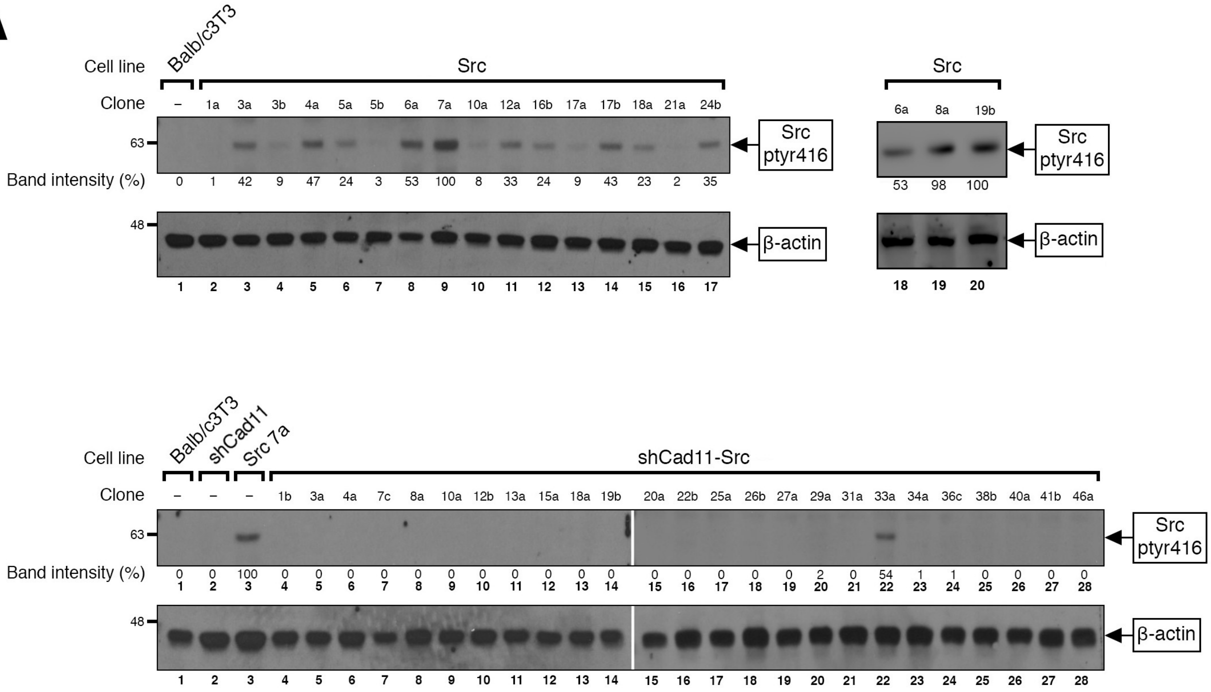

B

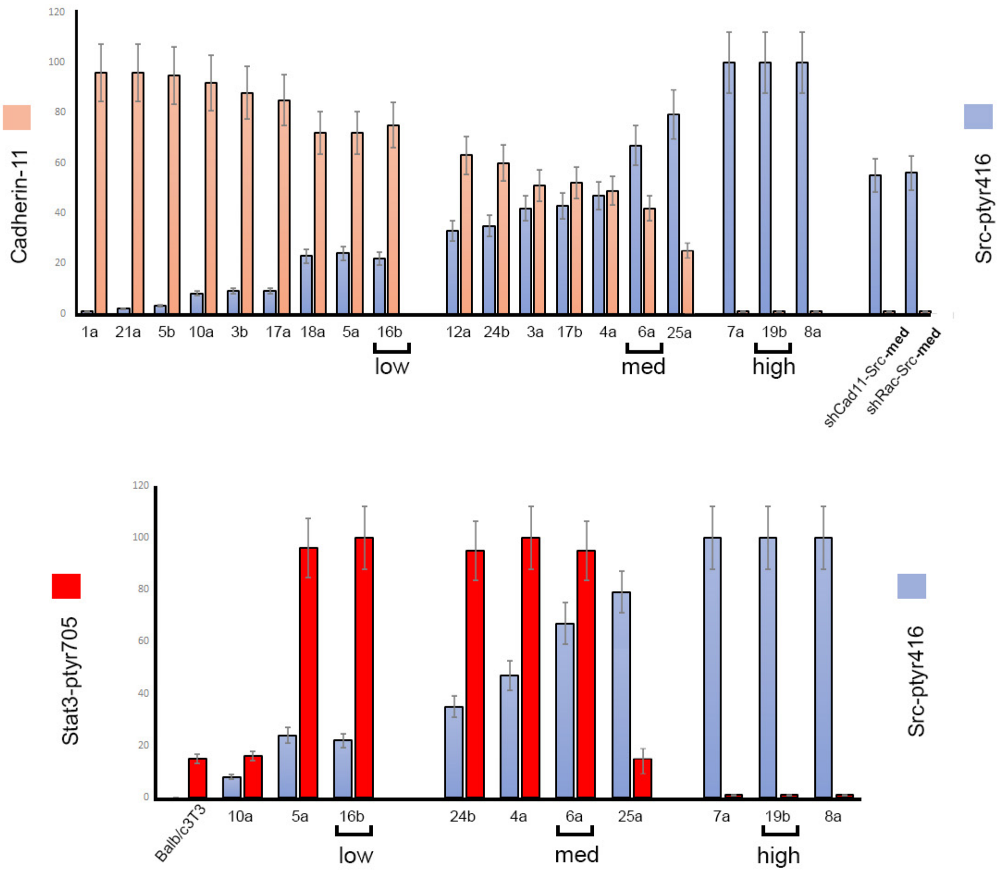
Cadherin-11
Src-ptyr416
0
20
40
60
80
100
120
1a
21a
5b
10a
3b
17a
18a
5a
16b
12a
24b
3a
17b
4a
6a
25a
7a
19b
8a
low
med
high
shCad11-Src-med
shRac-Src-med
Stat3-ptyr705
Src-ptyr416
Balb/c3T3
10a
5a
16b
24b
4a
6a
25a
7a
19b
8a
low
med
high

C

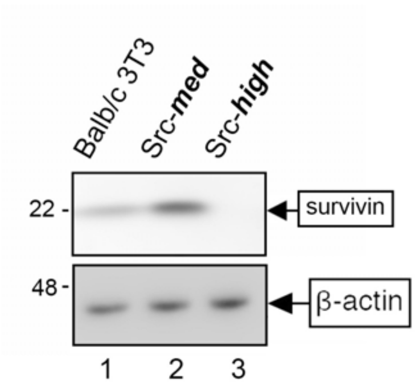
Balb/c 3T3
Src-med
Src-high
22 -
survivin
48 -
β-actin
1
2
3

Figure S1:

A. Expression of Src^{527F} in Balb/c3T3 mouse fibroblasts *vs* in shCad11

Top panel: Detergent extracts of individual Balb/c3T3 clones transduced with a Src^{527F}-expressing retroviral vector and selected for Hygromycin resistance, were probed for Src^{pY416} or β-actin as a loading control, as indicated. Numbers under the lanes of the Src^{pY416} panel refer to band intensities obtained through quantitation by image analysis and normalization to β-actin levels, with the peak value of Src^{527F} clone 7a (lane 9) taken as 100%. Numbers at the left refer to molecular weight markers.

Bottom panel: Balb/c3T3 cells in which Cad11 was knocked down using shRNA (shCad11) were infected with the same Src^{527F}-vector as above. Detergent cell extracts from individual clones obtained following hygromycin selection were probed for Src^{pY416}, or β-actin as a loading control, as above. Numbers under the lanes of the Src^{pY416} panel refer to band intensities obtained through quantitation by image analysis and normalization to β-actin levels, with the peak value of the same positive control as above (clone 7a) taken as 100% (lane 3). Numbers at the left refer to molecular weight markers.

B:

Top panel: **Activated Src^{527F} expression reduces Cadherin-11 levels in Balb/c3T3 cells in a quantitative manner**

Src^{pY416} and Cad11 levels were determined by Western blotting in detergent lysates of the clones described in A, top panel above. For a comparison, Src^{pY416} levels in shCad11-Src-***med*** and shRac-Src-***med*** clones are shown at the right. Note the graded decrease in Cadherin-11 levels with increasing Src^{527F}. Averages of three experiments ±SEM are shown.

Bottom panel: **High Src^{527F} expression dramatically reduces Stat3-ptyr705 levels**

The indicated lines were grown to densities of 100% confluence plus one day and Stat3-ptyr705 and Src^{pY416} levels determined by Western blotting. Note the increase in Stat3-ptyr705 in cells expressing low or medium Src^{527F} levels, and the dramatic Stat3-ptyr705 drop in lines expressing high Src^{527F}. Averages of three experiments ±SEM are shown.

C: Lysates from Balb/c3T3, Src-***med*** and Src-***high*** cells were probed for survivin or tubulin as a loading control. Note the reduction in surviving levels in Src-***high*** cells, compared with Src-***med***.

Knockdown of Cad11 in Src-*med* cells reduces Stat3-ptyr705

To further confirm the Cad11 requirement for Stat3 activation by Src^{527F}, we also performed the reverse experiment: Cad11 was knocked down with a retroviral vector with puromycin-resistance selection in Src-***med*** cells, which were previously shown to express low amounts of Cad11, due to Src expression (see Materials and Methods) (12). Following selection with puromycin, stable clones were picked and expanded into lines. Consistent with findings from Src^{527F}-expressing, Cad11 deficient lines above (Fig. S1A, bottom panel), it was not possible to downregulate Cad11 even further in lines with high Src^{527F}. As shown in Fig. S2 below, Stat3-ptyr705 levels increased with density in Src-***med*** cells (lanes 6-10) and the parental Balb/c3T3 (lanes 1-5). Interestingly, Src-***med***-shCad11 cells had lower Stat3-tyr705 phosphorylation at all densities examined (lanes 11-15) compared to Src-***med*** cells. This result further indicates that Cad11 may be required for Src-mediated, Stat3-ptyr705 phosphorylation; that is, there may be a fine Src/Cad11 balance required to achieve maximal Stat3-ptyr705 levels.

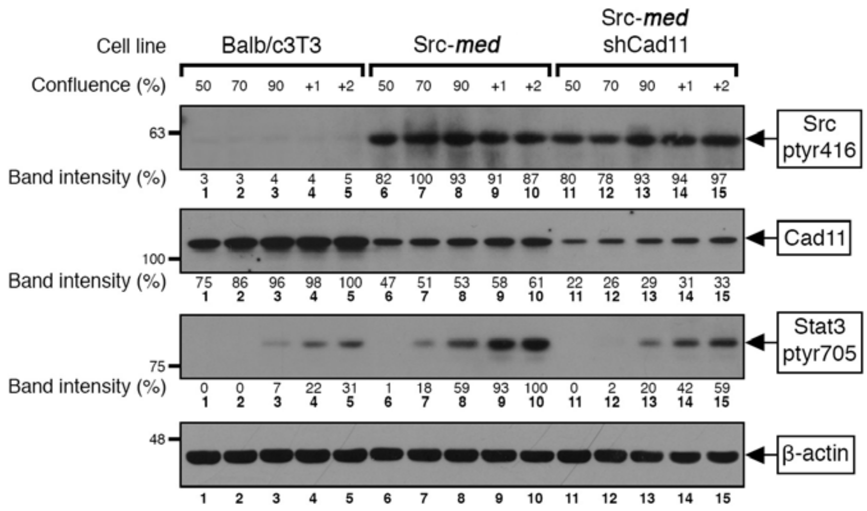

Figure S2: Knockdown of Cad11 in Balb-Src^{527F}-*med* cells reduces Stat3-ptyr705

Parental Balb/c3T3 (lanes 1-5), Src-***med*** (lanes 6-10), and Src-***med***-shCad11 (lanes 11-15) cells were grown to densities of 50% to 2 days post-confluence. Detergent cell extracts were probed for Stat3-ptyr705, Cad11, Src^{pY416}, or β-actin as a loading control, as indicated. Numbers immediately under the lanes of the Stat3-ptyr705 and Cad11 panels refer to band intensities obtained through quantitation by image analysis and normalized to β-actin levels, with the peak values of each taken as 100%. Numbers at the left refer to molecular weight markers.

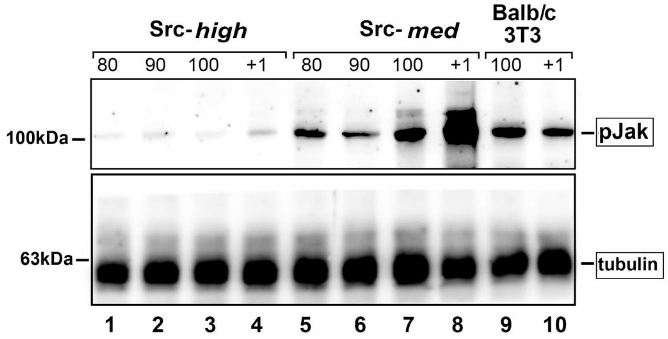

Fig. S3: High Src^{527F} expression results in a reduction of Jak-ptyr1022/1023 (p-Jak) levels.

We examined the effect of Src^{527F} expression and gp130 downregulation upon Jak-tyr1022/1023 phosphorylation (p-Jak) in detergent cell lysates of Src-***high*** (lanes 1-4), Src-***med*** (lanes 5-8) or the parental Balb/c3T3 cells (lanes 9-10) grown to different densities, by Western blotting, using tubulin as a loading control.

As shown above, there is an increase in p-Jak levels upon expression of intermediate Src^{527F} levels (lanes 5-8), compared to the parental Balb/c3T3 (lanes 9-10), which was more pronounced at higher densities (lane 8), in agreement with previous observations (46). Interestingly however, Src-***high*** cells displayed very low p-Jak levels, much lower than the parental Balb/c3T3 (lanes 1-4 *vs* 9-10). Therefore, the high IL6 secreted by the Src-***high*** cells (Fig. 4B) does not translate into high p-Jak, apparently due to the fact that gp130 receptor levels are very low in the absence of sufficient levels of Cad11.

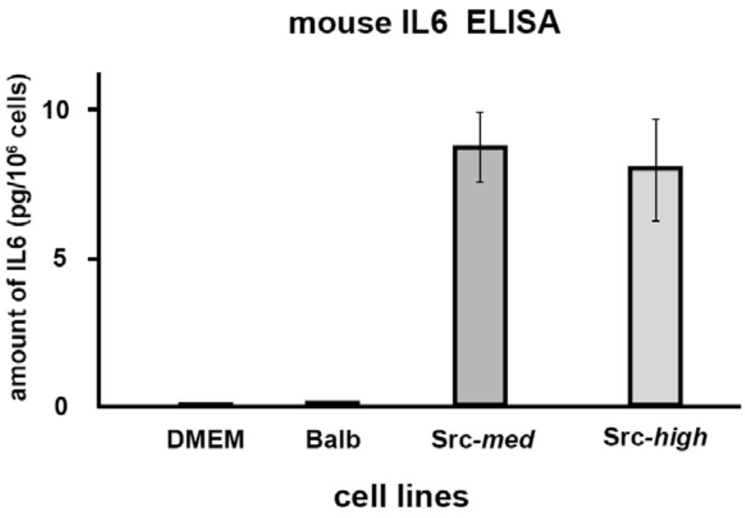

Figure S4: Src-*high* cells do secrete IL6 in their medium

Balb/c3T3 and derivatives were grown to 100% confluence on 10cm tissue culture plates. The medium was then changed to 3ml DMEM supplemented with 10% fetal bovine serum and cells incubated overnight. This "conditioned" medium was collected and filtered through a 0.22 µm filter. IL6 levels were subsequently examined using an enzyme-linked immunosorbent assay (ELISA), probing for mouse IL6, following the manufacturer's protocol (R&D Systems, DY406). Briefly, the wells of a 96-well plate were coated with rat anti-mouse IL-6 antibody reconstituted in PBS, overnight at room temperature. 100 µl of conditioned medium was then added to the wells and the plate incubated for 2 hours at room temperature. Duplicates of each sample were analyzed. The conditioned medium was subsequently aspirated, the wells washed and biotinylated goat anti-mouse IL-6 antibody was added, for 2 hours. Following washing, the wells were treated with Streptavidin conjugated to horseradish peroxidase for 20 minutes. For detection, 100 µl of the substrate solution (1:1 mixture of H_2O_2 and Tetramethylbenzidine) were added to the wells for 20 minutes. Absorbance values were then obtained for wavelengths of 450 and 540 nm. For quantitation of IL-6 levels, the absorbance values were compared to a standard curve obtained by running the recombinant mouse IL-6 standards (provided in the kit) with concentrations ranging from 15.6-1.000 pg/mL alongside the samples.

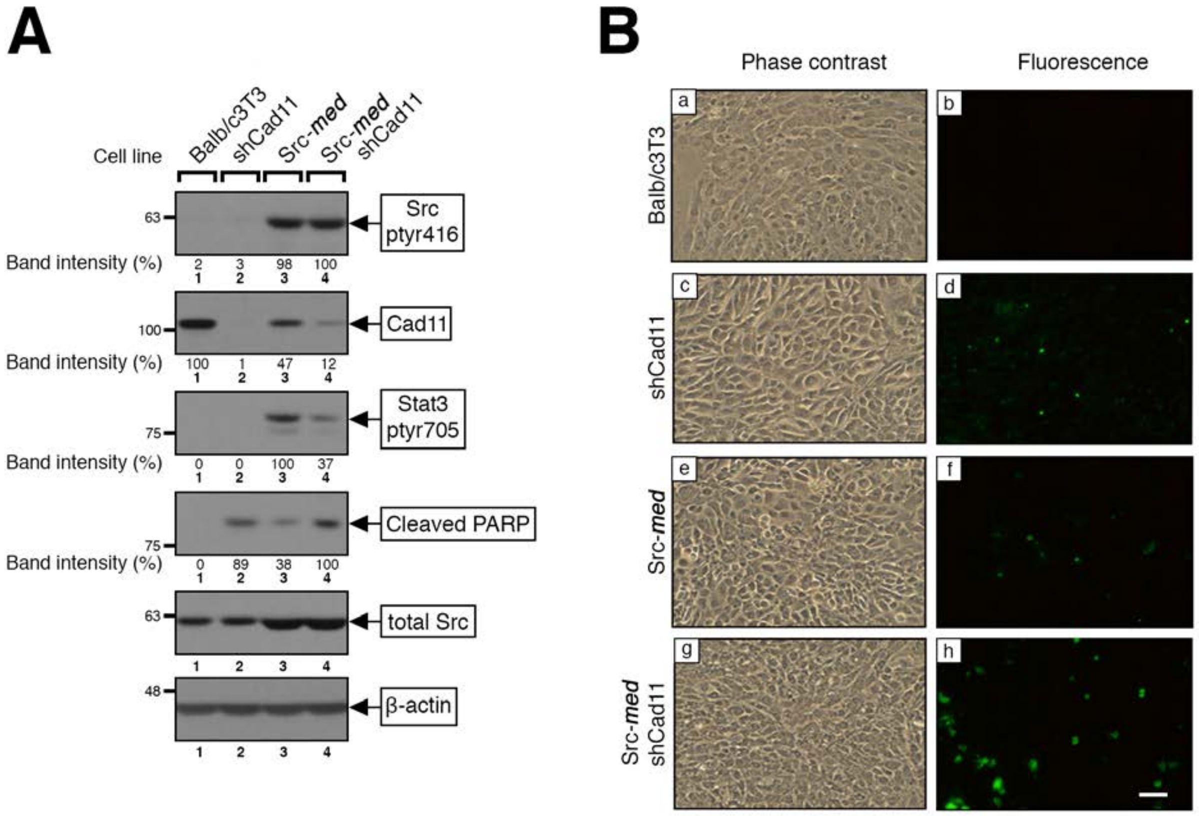

Figure S5: Knockdown of Cad11 in Src-*med* cells promotes apoptosis

A. Parental Balb/c3T3 (lane 1), shCad11 (lane 2), Src-***med*** (lane 3), and Src-***med***-shCad11 (lane 4) cells were grown to 2 days post-confluence. Detergent cell extracts were probed for Src^{pY416}, Cad11, Stat3-ptyr705, cleaved PARP, total Src, or β-actin as a loading control, as indicated. Numbers under the lanes of the upper panels refer to band intensities obtained through quantitation by image analysis and normalized to β-actin levels, with the peak values of Src-***med***-shCad11 (lane 4) for Src^{pY416} and cleaved PARP, Balb/c3T3 (lane 1) for Cad11, and Src-***med*** (lane 3) for Stat3-ptyr705 taken as 100%. Numbers at the left refer to molecular weight markers.

B. Balb/c3T3 (panels a, b), shCad11 (panels c, d), Src-***med*** (panels e, f), and Src-***med***-shCad11 (panels g, h) cells were grown to 1 day post-confluence and apoptosis examined by TUNEL staining. Cells were photographed under phase contrast and fluorescence illumination. Bar: 100 µm.

Note that Src-***med***-shCad11 cells expressed higher levels of cleaved PARP (lane 4) than Src-***med*** (lane 3) or the parental Balb/c3T3 (lane 1), and displayed higher levels of TUNEL staining than Src-***med*** cells (panels h *vs* d), indicating that Cad11 is required for survival of Src^{527F}-expressing cells.

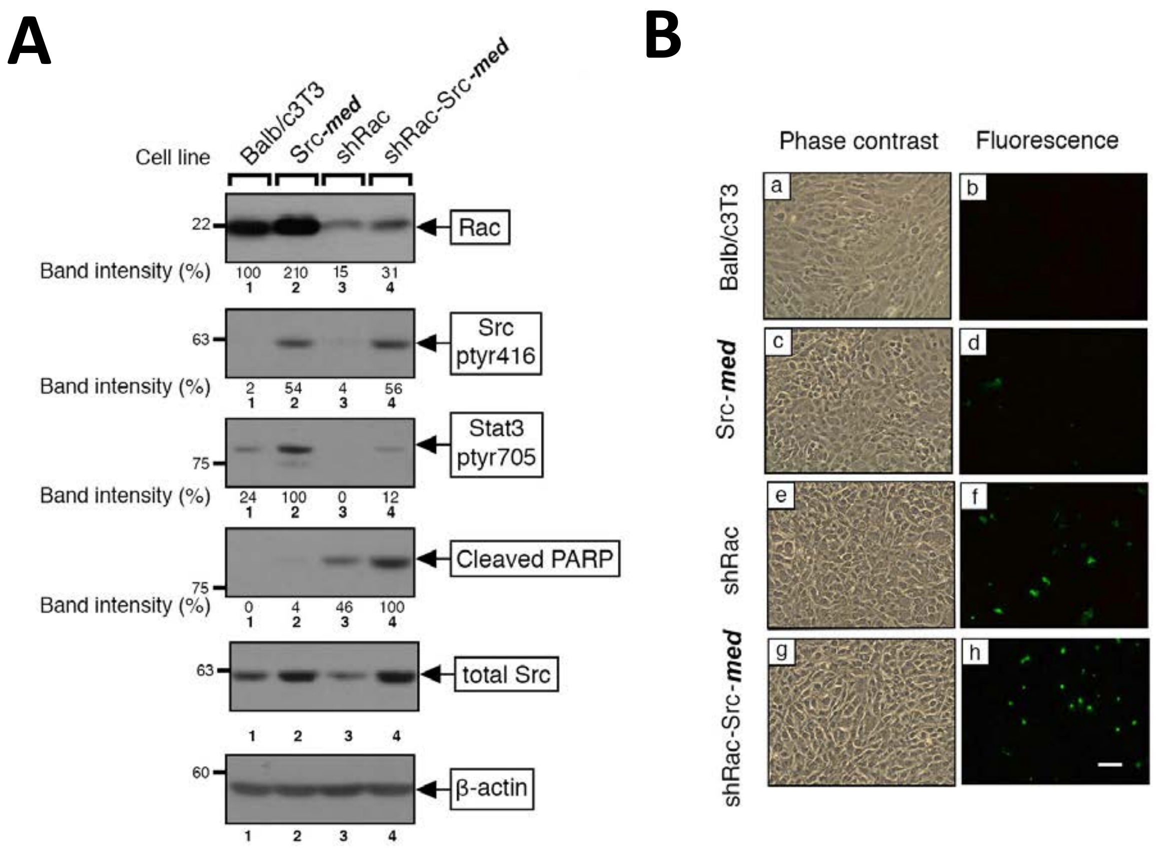

Figure S6: Expression of Src^{527F} in shRac cells promotes apoptosis

A: Parental Balb/c3T3 (lane 1), Src-***med*** (lane 2), shRac (lane 3), and shRac-Src-***med*** (lane 4) cells were grown to 2 days post-confluence. Detergent cell extracts were probed for Src^{pY416}, total Src, Rac, Stat3-ptyr705, cleaved PARP, or β-actin as a loading control, as indicated. Numbers under the lanes refer to band intensities obtained through quantitation by image analysis and normalization to β-actin levels. Numbers at the left refer to molecular weight markers. (Panels Rac, Src^{Y416}, Stat3-ptyr705, total Src and actin are from Fig. 3B).

B: Balb/c3T3 (panels a, b), Src-med (panels c, d), shRac (panels e, f), and shRac-Src-***med*** (panels g, h) cells were grown to densities of 2 days post-confluence and apoptosis was examined by TUNEL staining. Cells were photographed under phase contrast or fluorescence illumination. Bar: 100 µm.

Note that shRac-Src-***med*** cells expressed higher levels of cleaved PARP (lane 4) than Src-***med*** (lane 2) or the parental Balb/c3T3 (lane 1), and displayed higher levels of TUNEL staining than Src-***med*** cells (panels h *vs* d), indicating that Rac is required for survival of Src^{527F}-expressing cells.

A

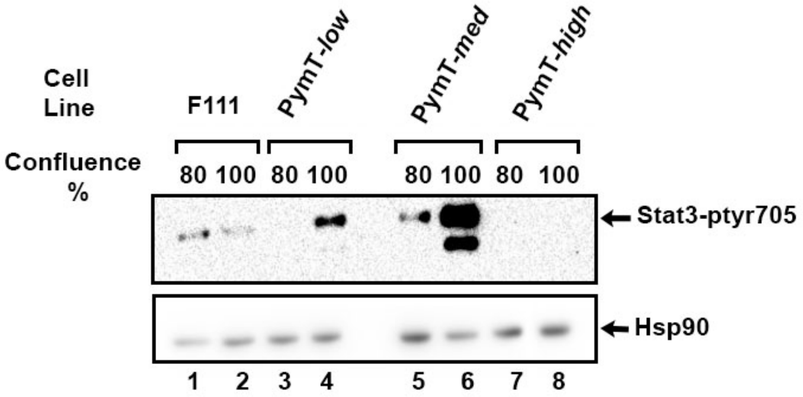
Cell Line
F111
PymT-low
PymT-med
PymT-high
Confluence %
80 100 80 100 80 100 80 100
Stat3-ptyr705
Hsp90
1 2 3 4 5 6 7 8

B

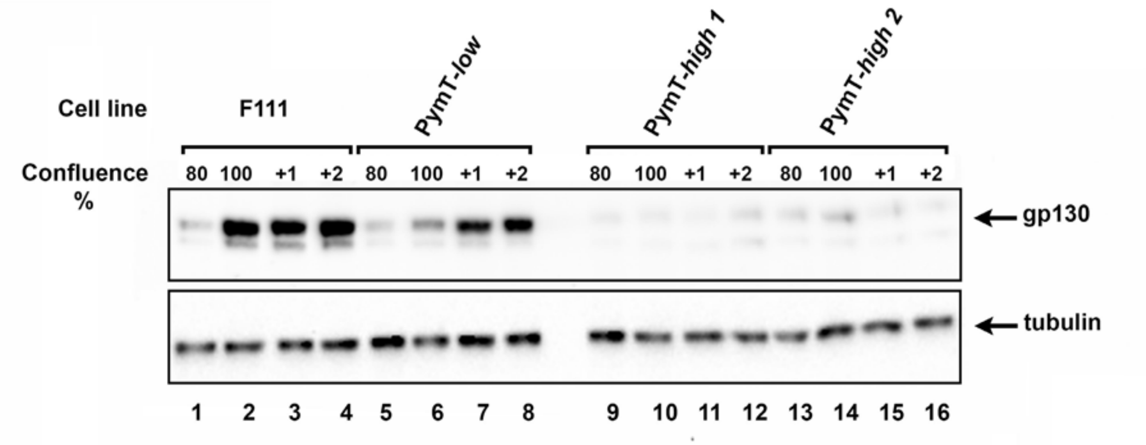
Cell line
F111
PymT-low
PymT-high 1
PymT-high 2
Confluence %
80 100 +1 +2 80 100 +1 +2 80 100 +1 +2 80 100 +1 +2
gp130
tubulin
1 2 3 4 5 6 7 8 9 10 11 12 13 14 15 16

C

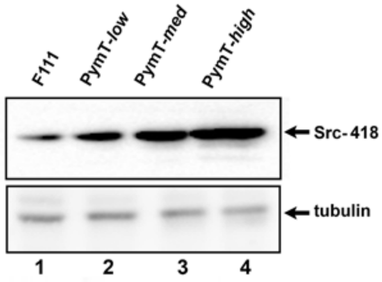
F111
PymT-low
PymT-med
PymT-high
Src-418
tubulin
1 2 3 4

Figure S7: Expression of high levels of the middle tumor antigen of polyoma virus (PymT) downregulates Stat3-ptyr705 and gp130.

A: PymT was expressed by transfection in Rat F111 fibroblasts under control of its native promotor (18). Clones expressing low (lanes 3, 4) medium (lanes 5, 6) or high (lanes 7, 8) PymT levels were grown to densities of 80% or 100% of confluence. Detergent cell extracts were then probed for Stat3-ptyr705 or Hsp90 as a loading control, as indicated (see Materials and Methods). Note the absence of Stat3-ptyr705 in PymT-***high*** cells (lanes 7, 8).

B: Extracts from F111 (lanes 1-4), or PymT-***low*** (lanes 5-8), or two clones expressing high PymT levels (PymT-***high-1***, lanes 9-12 and PymT-***high-2***, lanes 13-16) were grown to the indicated densities and detergent lysates probed for gp130 or β-tubulin as a loading control. Note the dramatic reduction in gp130 in cells expressing high PymT levels (lanes 9-16 *vs* 1-4).

C: Extracts from F111 (lane 1), or PymT-***low*** (lane 2), PymT-***med*** (lane 3) or PymT-***high*** (lane 4) cells grown to 100% confluence, were probed for Src-ptyr418 or β-tubulin as a loading control, as indicated.

Table S1: shRNA sequences

Target shRNA	OligoID	Sequence
shCad11-1	Open Biosystems V2MM-66004	TGCTGTTGACAGTGAGC sense: GACGTGAGAACATCATAACCTAT loop: TAGTGAAGCCACAGATGTA antisense: ATAGGTTATGATGTTCTCACGG - TGCCTACTGCCTCGGA
shCad11-2	Open Biosystems V2MM-75136	TGCTGTTGACAGTGAGC sense: GCGCCAACAGCCCAATAAGGTAT loop: TAGTGAAGCCACAGATGTA antisense: ATACCTTATTGGGCTGTTGGCA-TGCCTACTGCCTCGGA
shCad11-3	Open Biosystems V2MM-81396	TGCTGTTGACAGTGAGC sense: GCGGTATTCAATTGATCGTCATA loop: TAGTGAAGCCACAGATGTA antisense: TATGACGATCAATTGAATACCT-TGCCTACTGCCTCGGA
shCad11-4	Open Biosystems V2MM-66370	TGCTGTTGACAGTGAGC sense: GAGCACTCTCCAACCAGCCAATA loop: TAGTGAAGCCACAGATGTA antisense: TATTGGCTGGTTGGAGAGTGCCTGCC-TACTGCCTCGGA
shRac	Open Biosystems V2MM-7967	TGCTGTTGACAGTGAGCG sense: AGGCGTTGAGTCCATATTTAAA loop: TAGTGAAGCCACAGATGTA antisense: TTTAAATATGGACTCAACGCCC - TGCCTACTGCCTCGGA
